# Automatic segmentation of uterine endometrial cancer on multi-sequence MRI using a convolutional neural network

**DOI:** 10.1038/s41598-021-93792-7

**Published:** 2021-07-14

**Authors:** Yasuhisa Kurata, Mizuho Nishio, Yusaku Moribata, Aki Kido, Yuki Himoto, Satoshi Otani, Koji Fujimoto, Masahiro Yakami, Sachiko Minamiguchi, Masaki Mandai, Yuji Nakamoto

**Affiliations:** 1 Department of Diagnostic Imaging and Nuclear Medicine, Kyoto University Graduate School of Medicine, 54 Kawahara-cho, Shogoin, Sakyoku, Kyoto, 606-8507 Japan; 2 Department of Radiology, Kobe University Graduate School of Medicine, 7-5-2 Kusunoki-cho, Chuo-ku, Kobe, 650-0017 Japan; 3 Preemptive Medicine and Lifestyle-Related Disease Research Center, Kyoto University Hospital, 54 Kawahara-cho, Shogoin, Sakyoku, Kyoto, 606-8507 Japan; 4 Department of Real World Data Research and Development, Graduate School of Medicine, Kyoto University, 54 Kawahara-cho, Shogoin, Sakyoku, Kyoto, 606-8507 Japan; 5 Department of Diagnostic Pathology, Kyoto University Graduate School of Medicine, 54 Kawahara-cho, Shogoin, Sakyoku, Kyoto, 606-8507 Japan; 6 Department of Gynecology and Obstetrics, Kyoto University Graduate School of Medicine, 54 Kawahara-cho, Shogoin, Sakyoku, Kyoto, 606-8507 Japan

**Keywords:** Cancer imaging, Gynaecological cancer, Endometrial cancer

## Abstract

Endometrial cancer (EC) is the most common gynecological tumor in developed countries, and preoperative risk stratification is essential for personalized medicine. There have been several radiomics studies for noninvasive risk stratification of EC using MRI. Although tumor segmentation is usually necessary for these studies, manual segmentation is not only labor-intensive but may also be subjective. Therefore, our study aimed to perform the automatic segmentation of EC on MRI with a convolutional neural network. The effect of the input image sequence and batch size on the segmentation performance was also investigated. Of 200 patients with EC, 180 patients were used for training the modified U-net model; 20 patients for testing the segmentation performance and the robustness of automatically extracted radiomics features. Using multi-sequence images and larger batch size was effective for improving segmentation accuracy. The mean Dice similarity coefficient, sensitivity, and positive predictive value of our model for the test set were 0.806, 0.816, and 0.834, respectively. The robustness of automatically extracted first-order and shape-based features was high (median ICC = 0.86 and 0.96, respectively). Other high-order features presented moderate-high robustness (median ICC = 0.57–0.93). Our model could automatically segment EC on MRI and extract radiomics features with high reliability.

## Introduction

Endometrial cancer (EC) is the most common gynecological malignant tumor in developed countries, and its incidence rate has been increasing over the past decades^1^. Surgery is the main treatment for patients with EC. Magnetic resonance imaging (MRI) plays an essential role in surgical planning because it can provide information on the degree of myometrial invasion, presence of cervical stromal invasion, lymph node metastases, and extra-uterine spread^2^. While hysterectomy and bilateral salpingo-oophorectomy are the basic treatments for EC, pelvic and para-aortic lymphadenectomy are considered depending on the risk of recurrence^3,4^. There are several predictors of recurrence, including age, tumor grade, International Federation of Gynecology and Obstetrics (FIGO) stage, and lymphovascular space invasion^1^. However, many of these factors can be evaluated only after surgical treatment. Accurate preoperative risk stratification methods are needed to realize personalized treatment according to the prognosis. There have been several studies in which the radiomics approach was used for preoperative noninvasive risk stratification of EC using MRI^5–10^. Radiomics aims to extract and analyze numerous high‐dimensional quantitative features from medical images and is now widely used in tumor research^11^. Tumor segmentation is usually necessary when performing these studies. However, manual segmentation is not only labor-intensive and time-consuming but may also be subjective. Development of accurate automated segmentation methods is highly desirable.

U-net is a fully convolutional neural network architecture originally designed for segmentation of biomedical images and has shown promising results in segmentation of medical images^12^. The advantage of using U-net is that it it not necessary to manually create imaging features for segmentation. U-net has been applied not only to the segmentation of organs such as the breast, prostate, and uterus, but also to various diseases, including acute cerebral infarction, aortic dissection, acute pulmonary embolism, hepatocellular carcinoma, prostate cancer, and uterine cervical cancer^13–26^. One recent report demonstrated automatic segmentation of EC on MRI with 3D U-net^27^.However, the accuracy of segmentation has been variable and the robustness of radiomics features other than tumor volume has not been investigated.

The purpose of this research was to achieve automatic segmentation of EC on MRI with a convolutional neural network. We applied U-net and performed hyperparameter tuning for segmentation of EC and evaluated the effect of using multi-sequence images as input data. We also evaluated the robustness of the automatically extracted radiomics features.

## Results

### Clinical characteristics

The patient characteristics are presented in Supplementary Table S1. There was no significant difference in age, histological grade, FIGO stage, or frequency of deep myometrial invasion between the training and test datasets.

### Dice loss in our model and conventional U-net for five-fold cross-validation

The Dice losses in our models with input data for each MRI sequence are presented in Table 1. The model with multi-sequence images (T2-weighted image: T2WI, diffusion-weighted image: DWI, and apparent diffusion coefficient map: ADC map) as input data achieved the lowest Dice loss for both the training and validation sets. The Dice losses for the model with input data of multi-sequence images trained using different batch sizes are shown in Table 2. Given that the Dice loss improved by implementing larger batch sizes, our final model adopted a batch size of 176 (the mean and standard deviation of Dice loss for the training set and the five-fold cross-validation set was 0.159 ± 0.015 and 0.231 ± 0.033, respectively). The learning curve of our final model with five-fold cross-validation is presented in Supplementary Fig. S1-5.

**Table 1:** Dice losses for the models with each MRI sequence as the input data for five-fold cross-validation.

	T2WI	DWI	ADC	Multi-sequence
Train	0.192 ± 0.005	0.213 ± 0.009	0.237 ± 0.012	0.159 ± 0.015
Validation	0.298 ± 0.042	0.346 ± 0.053	0.431 ± 0.048	0.231 ± 0.033

Data are presented as the mean and standard deviation of five cross-validation models.

*Train*: train loss, *Validation*: validation loss, *ADC*: apparent diffusion coefficient map, *DWI*: diffusion-weighted image, *T2WI*: T2-weighted image.

**Table 2:** Dice losses for our model trained using different batch sizes with multi-sequence images.

Batch size	11	22	44	88	176 (final model)
Train	0.255 ± 0.005	0.194 ± 0.003	0.173 ± 0.009	0.162 ± 0.004	0.159 ± 0.015
Validation	0.592 ± 0.229	0.286 ± 0.034	0.251 ± 0.028	0.247 ± 0.027	0.231 ± 0.033

Data are presented as the mean and standard deviation.

*Train*: train loss, *Validation*: validation loss.

The mean and standard deviation of Dice loss for the conventional U-net model with a batch size of 11, 22, and 44 was 0.581 ± 0.233, 0.293 ± 0.026, and 0.267 ± 0.027, respectively, for five-fold cross-validation sets, which was worse than the Dice loss for our modified U-net^15^. Batch sizes of 88 and 176 could not be implemented in the conventional U-net model because of a lack of GPU memory.

### Evaluation of segmentation performance using the test dataset

**Figure 1:**
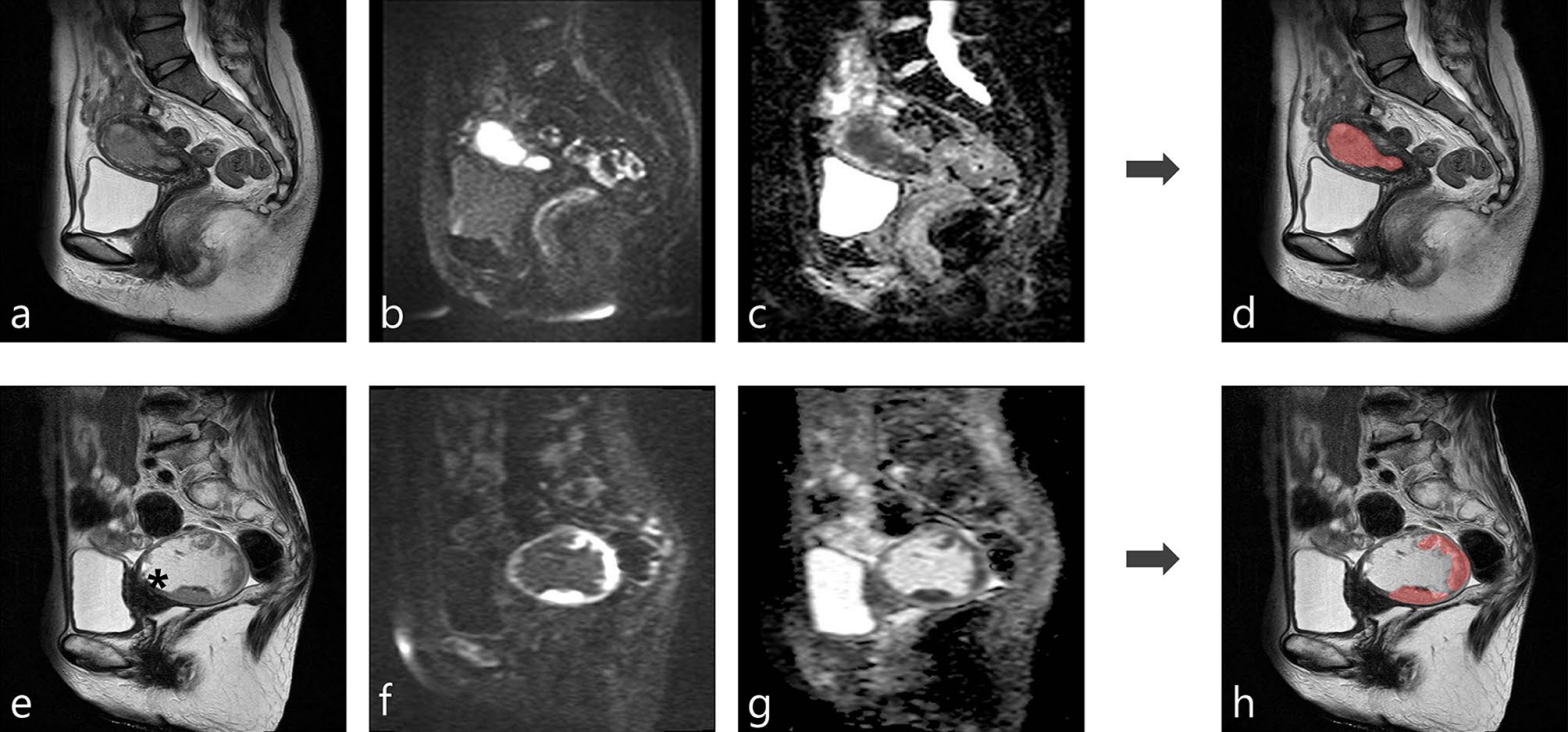
Two representative cases of automatic segmentation. (Case 1: a–d, Case 2: e–h). (**a﻿, e**): T2-weighted image. (**b**, **﻿f**): Diffusion-weighted image (b = 1000 s/mm^2^). (**c**, **﻿g**): Apparent diffusion coefficient map. (**d**, **﻿h**): Results of automatic segmentation of endometrial cancer overlaid on a T2-weighted image. The tumor was almost perfectly segmented in case 1 (Dice similarity coefficient, 0.925). The tumor was well segmented in case 2, despite the presence of hematometra (e:*) (Dice similarity coefficient, 0.808).

Table 3 shows the segmentation performance of our model using a batch size of 176 for the test datasets with each set of input data. The model with multi-sequence images as input data achieved the highest mean Dice similarity coefficient (DSC), sensitivity, and positive predictive value (PPV) (0.806, 0.816, and 0.834, respectively). There was no statistically significant difference in DSC, sensitivity, or PPV between the model with multi-sequence images and the model with T2WI (DSC, p = 0.67; sensitivity, *P*  = 0.12; PPV, *P*  = 0.15). However, there was a statistically significant difference between the model with multi-sequence images and the model with DWI or ADC map (all *P* < 0.001). The specificity and NPV were almost 1 for all models. In addition to mean DSC, to compare with the previous report, the median and interquartile range of DSC were also calculated in our model with multi-sequence images: median, 0.87; interquartile range, [0.80–0.90]^27^. Representative results for automatic segmentation with multi-sequence images are presented in Fig. 1.

**Table 3:** Segmentation performance of our model for the test datasets with each magnetic resonance image sequence as the input data.

	DSC	Sensitivity	Specificity	PPV	NPV
T2WI	0.798 ± 0.250	0.797 ± 0.261	1.000 ± 0.000	0.809 ± 0.234	1.000 ± 0.000
DWI	0.679 ± 0.207	0.694 ± 0.245	0.999 ± 0.001	0.684 ± 0.175	0.999 ± 0.001
ADC	0.557 ± 0.330	0.570 ± 0.369	0.999 ± 0.001	0.586 ± 0.305	0.999 ± 0.001
Multi	0.806 ± 0.155	0.816 ± 0.212	0.999 ± 0.001	0.834 ± 0.086	1.000 ± 0.000

Data are presented as the mean and standard deviation.

*ADC*: apparent diffusion coefficient map, *DSC*: Dice similarity coefficient, *DWI*: diffusion-weighted image, *Multi* multi-sequence images (T2WI, DWI, and ADC map), *NPV*: negative predictive value, *PPV* : positive predictive value, *T2WI*: T2-weighted image.

### Robustness of radiomics features

**Figure 2:**
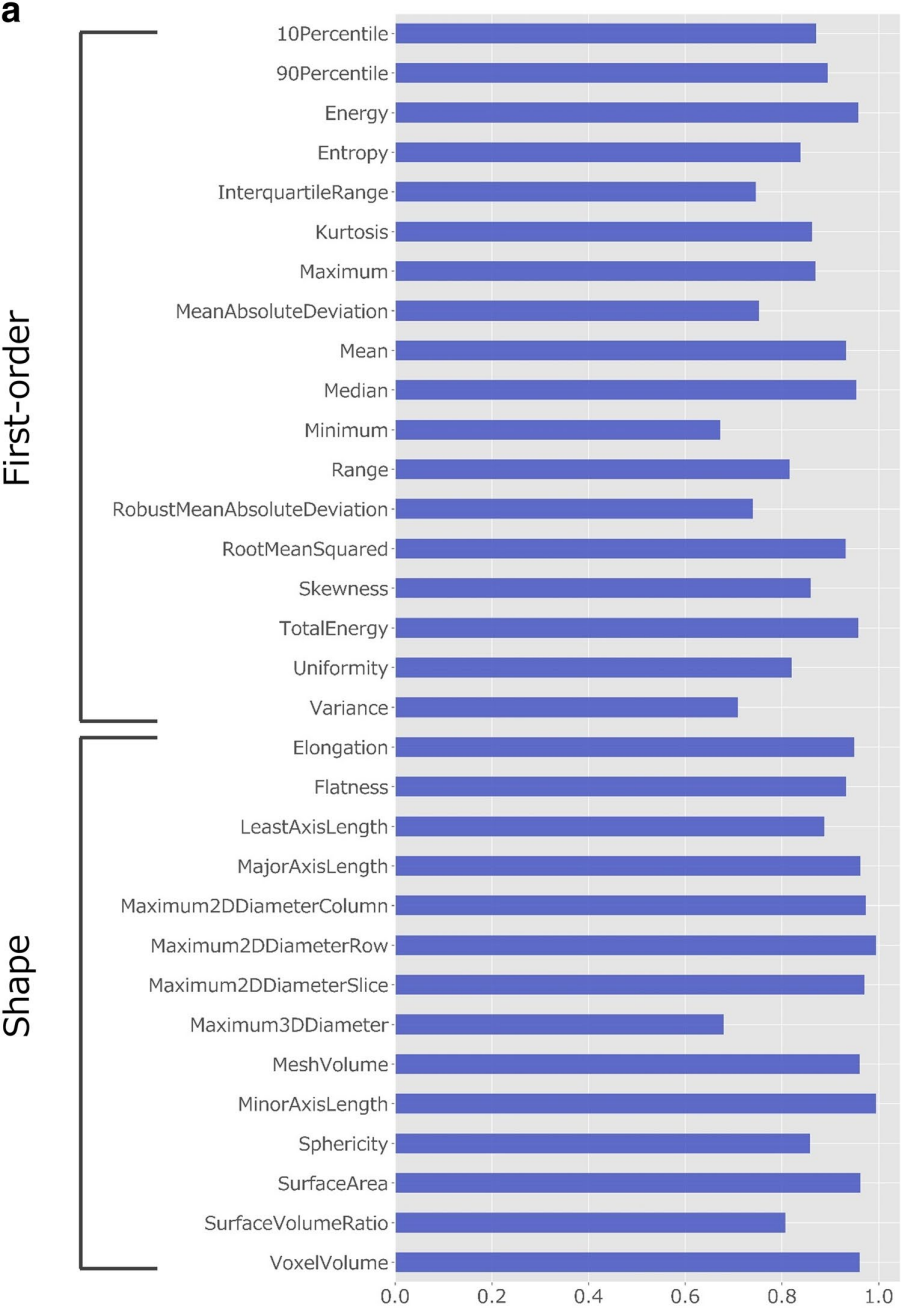

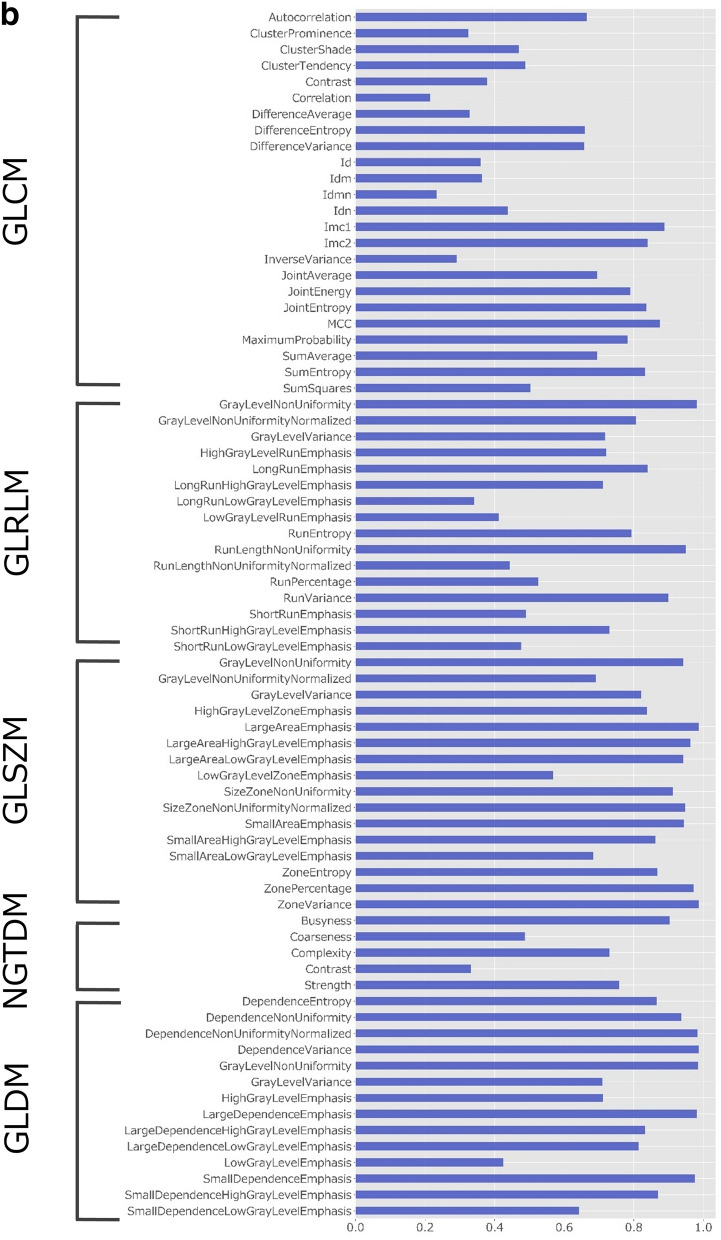
ICC values for radiomics features obtained by manual and automatic segmentation. (**a**) ICC values for first-order and shape-based features. (**b**) ICC values for features with high-order (GLCM, GLRLM, GLSZM, NGTDM, and GLDM-based) features. *GLCM*: gray-level co-occurrence matrix, *GLDM*: gray-level dependence matrix, *GLRLM*: gray-level run-length matrix, *GLSZM*: gray-level size zone matrix, *ICC*: intraclass correlation coefficient, *NGTDM*: neighboring gray tone difference matrix.

The intraclass correlation coefficient (ICC) values of the radiomics features obtained by manual and automatic segmentation are presented in Fig. 2. First-order and shape-based features showed good–excellent reliability (ICC, 0.75–0.99) except for minimum, robust mean absolute deviation, variance, and maximum 3D diameter (ICC, 0.67–0.74). Table 4 presents the median and interquartile range of ICC values per feature group. While textural features from gray level co-occurrence matrix (GLCM), gray level run length matrix (GLRLM), and neighboring gray tone difference matrix (NGTDM) showed moderate reliability, features from gray level size zone matrix (GLSZM) and gray level dependence matrix (GLDM) showed good reliability.

**Table 4:** Median and interquartile range for the ICC values per feature group.

Feature group	Median ICC	Interquartile range
first-order features	0.86	0.75–0.93
shape-based features	0.96	0.88–0.97
GLCM	0.57	0.36–0.79
GLRLM	0.72	0.48–0.83
GLSZM	0.93	0.83–0.96
NGTDM	0.73	0.41–0.83
GLDM	0.87	0.71–0.98

*GLCM* : gray-level co-occurrence matrix, *GLRLM*: gray-level run-length matrix, *GLSZM*: gray-level size zone matrix, *GLDM*: gray-level dependence matrix, *ICC*: intraclass correlation coefficient, *NGTDM*: neighboring gray tone difference matrix.

## Discussion

In this study, we performed automatic segmentation of EC on MRI with a convolutional neural network. Use of multi-sequence MR images as input data and larger batch sizes improved the accuracy of segmentation. Our final model achieved a mean DSC of 0.806 for the test dataset. Radiomics features obtained with our model demonstrated high reliability, especially for first-order and shape-based features.

To our knowledge, only one previous study has performed automatic segmentation of uterine EC^27^. The segmentation performance of EC by our model outperformed the previous model (median DSC 0.87 vs. 0.84 and 0.77 for two raters) with less variability; the likely reason for this is that the previous model used only contrast-enhanced T1-weighted image (T1WI) for segmentation whereas our model used multi-sequence images with ensemble learning. For the input data of the modified U-net, high accuracy was achieved using multi-sequence images, including T2WI, DWI, and ADC maps. Although EC usually shows high signal intensity on DWI because of its high cellularity, normal uterine endometrium also shows high signal intensity on DWI, and radiologists recognize the extent of EC in clinical practice by referring to various sequence images^28^. This could be the reason why the model with multi-sequence images showed higher accuracy than the other models. In a previous study of automatic segmentation of uterine cervical cancer on MRI, use of multi-sequence MR images (DWI with b = 0 and 1000 s/mm^2^ and an ADC map for the triple channel) as input data was beneficial for tumor segmentation^20^. The model developed in that study achieved a DSC of 0.82 for cervical cancer in the test dataset. Our model fell slightly short of this result, likely because the uterine body contains more structures that can interfere with segmentation, such as normal endometrium and retained fluid compared with the uterine cervix.

Our study also showed that a larger batch size contributed to improve segmentation performance. In general, it has been reported that a large batch size has an effect similar to that of using a small learning rate and is useful for accelerating learning^29^. However, there are very few reports on the relationship between segmentation accuracy and batch size. A previous report on segmentation of the uterus on MRI showed that a large batch size was useful for improving segmentation performance, which is consistent with our findings^15^. In this study, all MR image slices were used as input data with a view to clinical application. Given that only some of the slices contain tumor tissue, using a small batch size would increase the frequency of batches that do not or contain only very few slices of tumors as input data, making it difficult to train the model. We believe that this is the reason why segmentation performance was improved by using a large batch size. It is usually difficult to use a large batch size because image segmentation often requires a large amount of memory. One of the strengths of our research is that we could apply a batch size of 176 by using a graphic processing unit (GPU) with a large memory size of 96 GB. Even with this large GPU memory, the conventional U-net model could not apply a batch size of more than 44 because it applies 64 filters in the first convolution process^12^, which might explain why the segmentation performance of the conventional U-net model was worse than that of our model.

Many of the radiomics features extracted by our U-net model demonstrated good reliability in terms of first-order features, shape-based features, and features from GLSZM and GLDM. Automatically extracted radiomics features must be reliable for clinical application of these features as biomarkers. The robustness of radiomics features is reportedly affected by the type of tumor and its location as well as the software used for feature extraction^30,31^. Although no studies have examined the robustness of imaging features of EC, two reports have investigated uterine cervical cancer. In a study of the stability of radiomics features of uterine cervical cancer detected on T2WI using pyradiomics, it was found that the ICCs for features extracted by two independent radiologists were excellent (66.7% for first-order features, 100% for shape-based features, and 63.5% for features from the matrices)^32^. Another report on the robustness of radiomics features from the ADC map showed that the reproducibility of manually and automatically extracted features was low except for first-order features^20^. As mentioned earlier, it is difficult to make direct comparisons between the results of studies in cervical cancer with those in EC. However, the reliability of the radiomics features automatically extracted by our model was much higher than that reported previously, and for some features, the reliability was close to that extracted manually by radiologists.

There are some limitations to this study. First, it had a single-center retrospective design and used a single MR scanner vendor. A validation study using multicenter cases and multi-vendor MR scanners is needed to test the robustness and generalizability of our automatic segmentation model. Second, we did not use contrast-enhanced (CE) T1WI for automatic segmentation. Although adding CE T1WI to the input data may further improve segmentation accuracy, our model showed high segmentation performance without CE T1WI for the labels created by board-certified gynecologic radiologists referring to multiple sequences of images including CE T1WI. Besides, in clinical practice, not all patients with EC can undergo CE MRI because of renal dysfunction or allergy to contrast media. Even for these patients, our model allows for automatic segmentation of EC.

In conclusion, this study showed that our U-net model could perform accurate segmentation of EC on MRI. First-order, shape-based, and some high-order radiomics features can be extracted automatically with high reliability. Our model would make it possible to prepare a large number of region of interest (ROI) for EC with less effort, which leads to efficient medical image analysis using the radiomics approach and/or deep learning methods for risk stratification of EC.

## Methods

This single-center retrospective study was approved by Kyoto University Graduate School and Faculty of Medicine, Ethics Committee, and the requirement for written informed consent was waived (R1458-2). All methods were performed in accordance with the relevant guidelines and regulations.

### Patients

Two hundred patients pathologically diagnosed as EC who underwent pretreatment MRI between January 2004 and March 2017 were included in this study. These patients had previously been included in an as yet unpublished study of texture analysis with manual segmentation that aimed to identify prognostic risk factors for EC. One hundred and eighty patients were randomly selected from the 200 patients as the training dataset for training the model parameters and the other 20 as the test dataset to evaluate the performance of the final model. One board-certified radiologist (S.O. having 9 years’ experience) searched the clinical and pathological records for clinical information, including patient age, pathological diagnosis of the tumor, FIGO stage, and presence of deep myometrial invasion. Endometrioid carcinoma G1/G2 was categorized as a low-grade tumor, and other histological subtypes, such as endometrioid carcinoma G3 or serous carcinoma, were categorized as high-grade tumors.

### MRI protocol

MRI studies were performed using a 1.5-T unit (Symphony or Avanto; Siemens Health Care, Erlangen, Germany) or a 3.0-T unit (Trio, Skyra; Siemens Health Care) with a phased-array coil. Before the examination, 20 mg of scopolamine butylbromide (Buscopan; Nippon Boehringer Ingelheim, Tokyo, Japan) was administered intramuscularly to reduce motion artifact due to bowel peristalsis unless contraindicated. Routine MR sequences for uterine EC included three orthogonal planes of T2WI, axial and sagittal T1WI with or without fat suppression, axial or sagittal DWI, and axial or sagittal dynamic contrast-enhanced T1WI. The b-values applied for DWI had some variation: b = 0, 500, and 1000 s/mm^2^, and b = 0, 100, 500, and 1000 s/mm^2^. ADC values were calculated by fitting the signal intensities acquired from the different b-values to a mono-exponential model using the least-squares method. Dynamic CE T1WI was acquired at 20, 40, 60, 80, 100, 120, and 180 s after intravenous injection of the gadolinium contrast agent (Magnevist; Bayer Yakuhin Ltd, Osaka, Japan) at a dose of 0.2 mL/kg. The imaging parameters are presented in Supplementary Table S2.

### Image annotation

A board-certified gynecologic radiologist (Y.K. having 13 years’ experience) manually segmented the uterine ECs on each slice of the sagittal T2WI using a 3D Slicer (https://www.slicer.org/) by referring to all the images of other sequences and pathological reports. The other board-certified gynecologic radiologist (Y.M. having 13 years’ experience) confirmed the validity of the ROI in all cases. These ROIs were the gold standard for tumor segmentation.

### Image preprocessing

The field of view (FOV) was aligned for cases with a difference in the FOV between T2WI and DWI. The MR images were then resized to 512 × 512 pixels; the MR signal intensities of the images were normalized based on the following Eq. (1):

1 $$nSI = ~\frac{{\left( {SI - mean\_of\_SI} \right)}}{{\left( {3 \times SD\_of\_SI} \right)}}$$

where nSI is the normalized SI, mean_of_SI is the mean SI of the images, and SD_of_SI is the standard deviation (SD) of the SI of the images.

To evaluate the effect of a multi-sequence of MR images for improving segmentation accuracy, single-sequence images (one of T2WI, DWI, and the ADC map), and multi-sequence images (T2WI-DWI-ADC map for triple channel) were used as input data. Dynamic contrast-enhanced T1WI was not used because the imaging direction was different for each patient.

### Our U-net architecture

**Figure 3:**
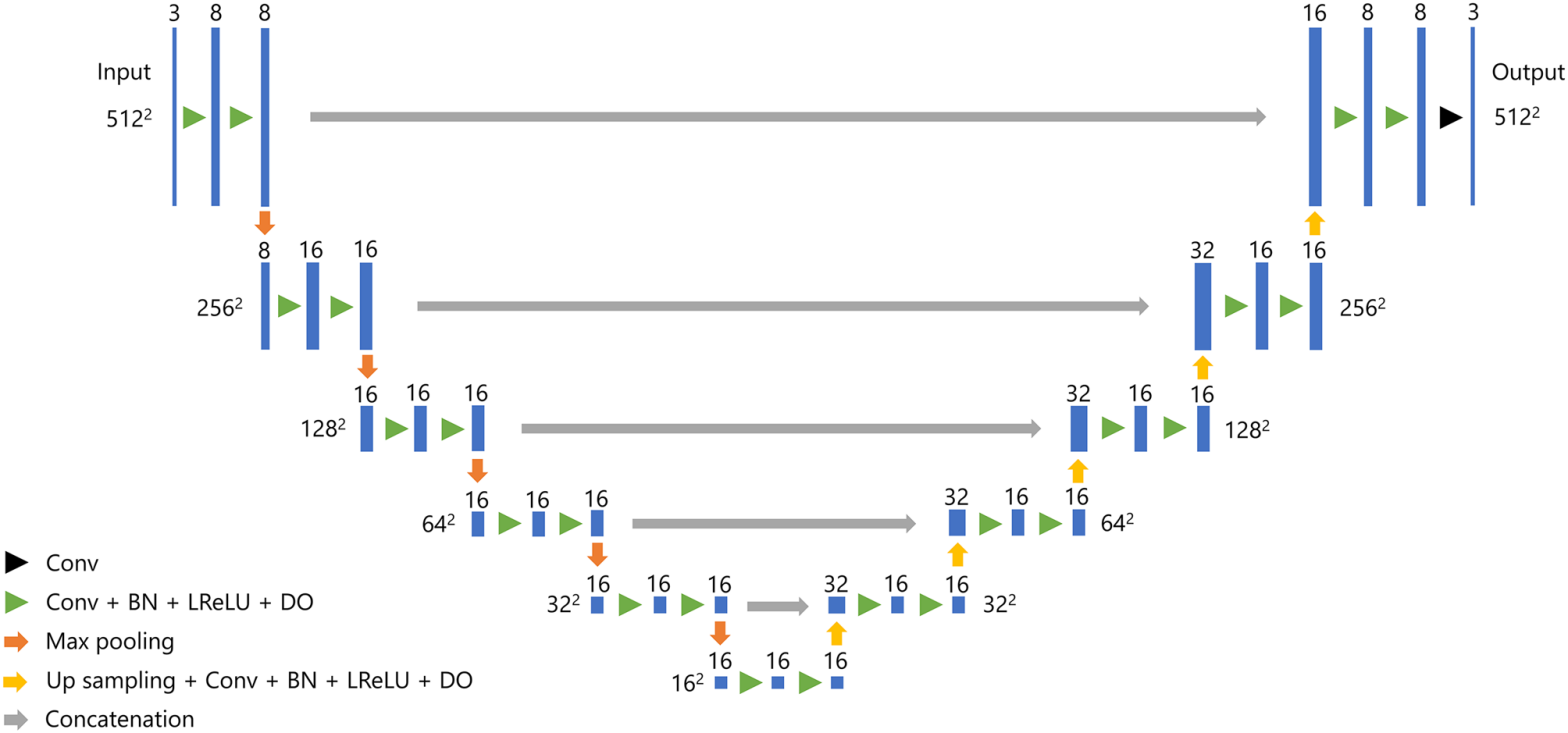
Our U-net architecture for segmentation of uterine endometrial cancer. *Conv*: convolution, *BN*: batch normalization, *LReLU*: Leaky Rectified Linear Unit, *DO* : dropout.

Our modified U-net architecture for segmentation of uterine EC is composed of five blocks, the detailed structure of which is presented in Fig. 3 and Supplementary Table S3. The Adam optimizer was used to train the neural network with the Dice loss function as the cost function. The initial learning rate was set to 0.005 and decreased to 0.0005 halfway through the training process. The dropout probability was set to 0.9%. To evaluate the effect of batch size, Dice loss with batches of 11, 22, 44, 88, and 176 were calculated. Five-fold cross-validation was performed with four-fifths of the patients used for training and one-fifth used for validation. The model was trained for 30 epochs for each cross-validation set. To prevent overfitting to the training dataset, we applied mix-up (β = 0.1) and random image cropping and patching (β = 0.3) as data augmentation methods^33–35^. For comparison, a conventional U-net model was trained for segmentation with batch sizes of 11, 22, and 44. The architecture of the conventional U-net is the same as that in a previous study of uterine segmentation^15^. Batch sizes of 88 and 176 could not be implemented in the conventional U-net model because of a lack of GPU memory.

Our model was built using Keras (version 2.3.1) and Tensorflow (version 1.15.0) and trained on a Linux workstation (Ubuntu version 18.04.4) with two NVIDIA Quadro RTX8000 GPUs with 48 GB memory (NVIDIA, Santa Clara, CA, USA).

### Evaluation of segmentation performance

**Figure 4:**
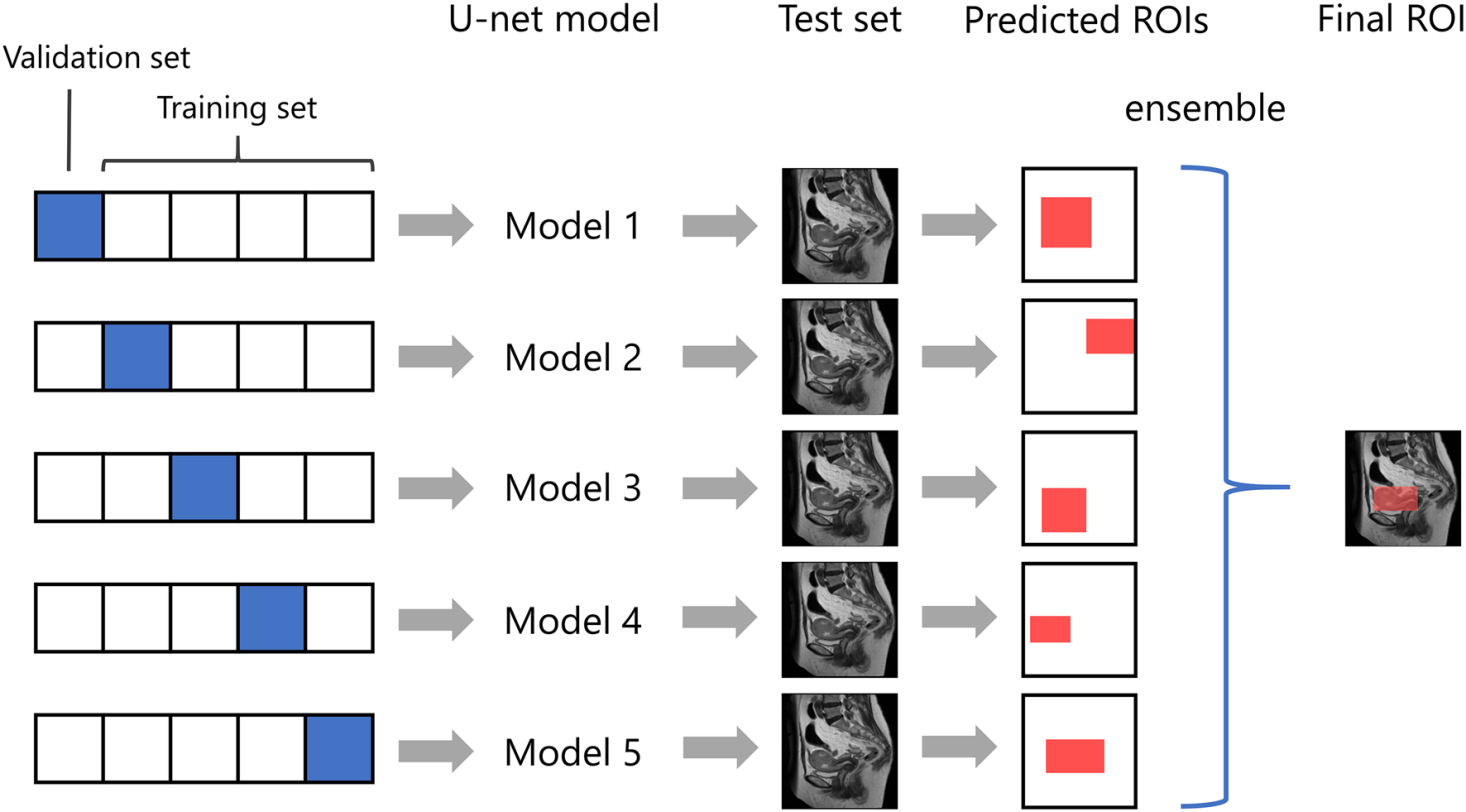
Overall workflow for predicting the area of endometrial cancer. *ROI*: region of interest.

For segmentation of uterine EC in the test datasets, an ensemble model of the five models trained by each cross-validation dataset was used. That is, the area predicted as uterine EC by more than two of the five models was determined to be the tumor area. The overall workflow for predicting the final ROI of the EC is presented in Fig. 4. The area predicted as a tumor within a 10% margin of each image was discarded because the uterus and EC do not usually exist on the edge of the MR images. The segmentation accuracy for the test datasets was evaluated using the DSC, sensitivity, specificity, and PPV, and negative predictive value (NPV) for each patient. DSC, sensitivity, specificity, PPV, and NPV were defined were defined by the following Eqs. (2–6).

2 $$DSC = \frac{{2\left| {T \cap P} \right|}}{{\left( {\left| T \right| + \left| P \right|} \right)}}$$

3 $$Sensitivity = \frac{{\left| {T \cap P} \right|}}{{\left| T \right|}}$$

4 $$Specificity = 1 - \frac{{\left| P \right| - \left| {T \cap P} \right|}}{{\left| I \right| - \left| T \right|}}$$

5 $$PPV = \frac{{\left| {T \cap P} \right|}}{{\left| P \right|}}$$

6 $$NPV = 1 - \frac{{\left| T \right| - \left| {T \cap P} \right|}}{{\left| I \right| - \left| P \right|}}$$

where |T|, |P|, and |I| denote the number of voxels for the true manual segmentation, the predicted segmentation, and the number of voxels of the three-dimensional MRI images, respectively.

The DSC represents the similarity between two sets of data. A DSC of 0 indicates no overlap whereas a DSC of 1 indicates perfect overlap between the two maps^36^.

### Extraction of radiomics features

The radiomics features of EC were extracted using Pyradiomics (version 3.0.1) software (https://www.radiomics.io/index.html) based on the volume-of-interest (VOI) from T2WI with manual and automatic segmentation by our U-net model. For discretization of pixel values, a fixed bin width of 0.0167 was adopted, which made the bin count around 120. The radiomics features calculated were first-order features (n = 18), shape-based features (n = 14), and features with GLCM (n = 24), GLRLM (n = 16), GLSZM (n = 16), NGTDM (n = 5), and GLDM (n = 14). A definition of each radiomics feature can be found online (https://pyradiomics.readthedocs.io/en/latest/features.html).

### Statistical analysis

Statistical analyses were performed using a commercially available software package (JMP version 12.2.0, SAS Institute Inc., Cary, NC, USA). The clinical characteristics of the training and test datasets were compared for age using the *t*-test. The distributions of histological grade and FIGO stage and the frequency of deep myometrial invasion were compared using a two-sided Fisher’s exact test. The DSC, sensitivity, and PPV of the test datasets with the different MRI sequences as input data were compared using the Wilcoxon signed-rank test. The reproducibility of radiomics features of EC with VOIs of manual and automatic segmentation was evaluated using the ICC, which was calculated using pingouin (version 0.3.8) software (https://pingouin-stats.org/). ICC values were interpreted as follows: < 0.5 poor, 0.5–0.75 moderate, 0.75–0.9 good, > 0.9 excellent^37^. A *P*-value < 0.05 was considered statistically significant. All *P*-values were adjusted for multiple comparisons using the Holm method^38^.

## Supplementary Information

Supplementary Information.
