# Supplementary material for: Automatic segmentation of uterine endometrial cancer on multi-sequence MRI using a convolutional neural network
Source: Sci Rep. 2021 Jul 14;11:14440. doi: 10.1038/s41598-021-93792-7 (PMC8280152; doi:10.1038/s41598-021-93792-7)
Supplement: Supplementary file 1 — Supplementary Information. [file 41598_2021_93792_MOESM1_ESM.pdf]

Automatic segmentation of uterine endometrial cancer on multi-sequence MRI using a convolutional neural network

Yasuhisa Kurata, MD PhD,[1] Mizuho Nishio*, MD PhD, [1,2] Yusaku Moribata, MD PhD, [1,3] Aki Kido, MD PhD,[1] Yuki Himoto, MD PhD,[1] Satoshi Otani, MD,[1] Koji Fujimoto, MD PhD,[4] Masahiro Yakami, MD PhD,[1,3] Sachiko Minamiguchi, MD PhD,[5] Masaki Mandai, MD PhD,[6] Yuji Nakamoto, MD PhD[1]

[1]Department of Diagnostic Imaging and Nuclear Medicine, Kyoto University Graduate School of Medicine, 54 Kawahara-cho, Shogoin, Sakyoku, Kyoto 606-8507, Japan

[2]Department of Radiology, Kobe University Hospital, 7-5-2 Kusunoki-cho, Chuo-ku, Kobe 650-0017, Japan.

[3]Preemptive Medicine and Lifestyle-Related Disease Research Center, Kyoto University Hospital, 54 Kawahara-cho, Shogoin, Sakyoku, Kyoto 606-8507, Japan

[4]Department of Real World Data Research and Development, Graduate School of Medicine, Kyoto University, 54 Kawahara-cho, Shogoin, Sakyoku, Kyoto 606-8507, Japan

[5]Department of Diagnostic Pathology, Kyoto University Graduate School of Medicine, 54 Kawahara-cho, Shogoin, Sakyoku, Kyoto 606-8507, Japan

[6]Department of Gynecology and Obstetrics, Kyoto University Graduate School of Medicine, 54 Kawahara-cho, Shogoin, Sakyoku, Kyoto 606-8507, Japan

Figure S1-5: The learning curve of our final model with five-fold cross-validation

loss: Training loss

val_loss: Validation loss

Figure S1

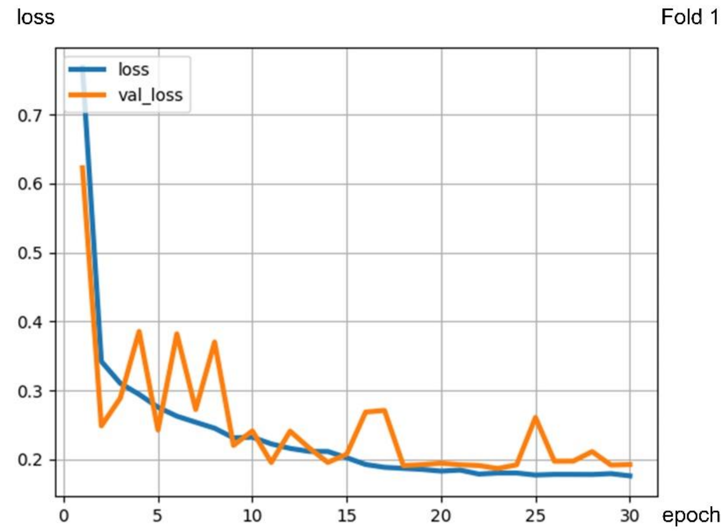

Figure S2

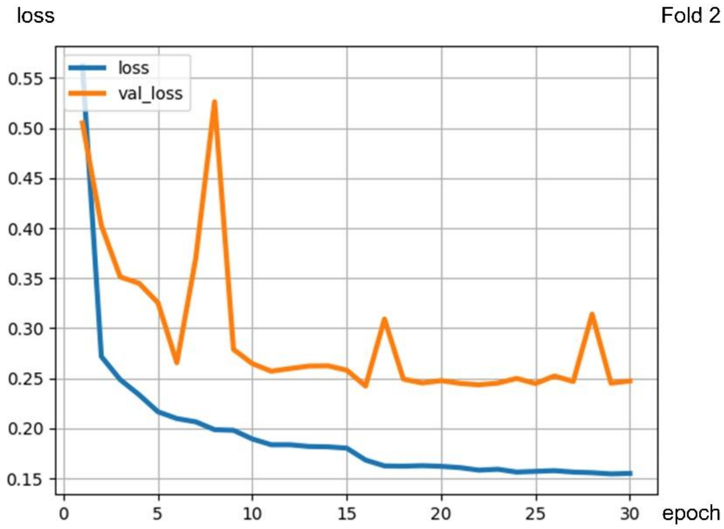

Figure S3

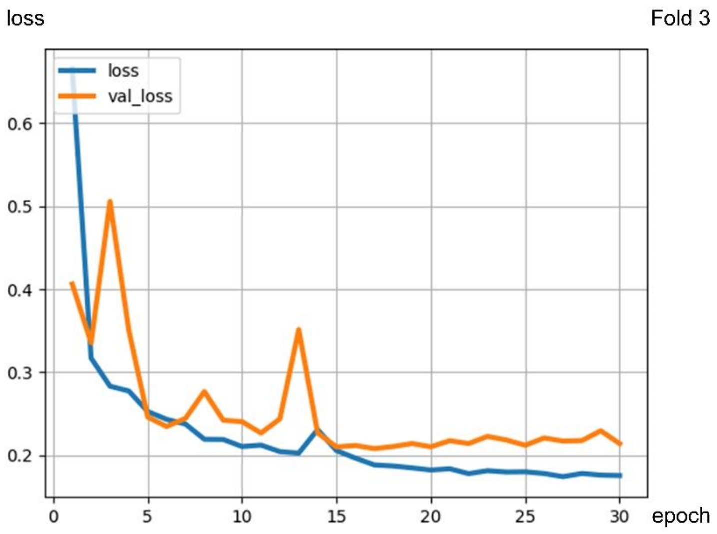

Figure S4

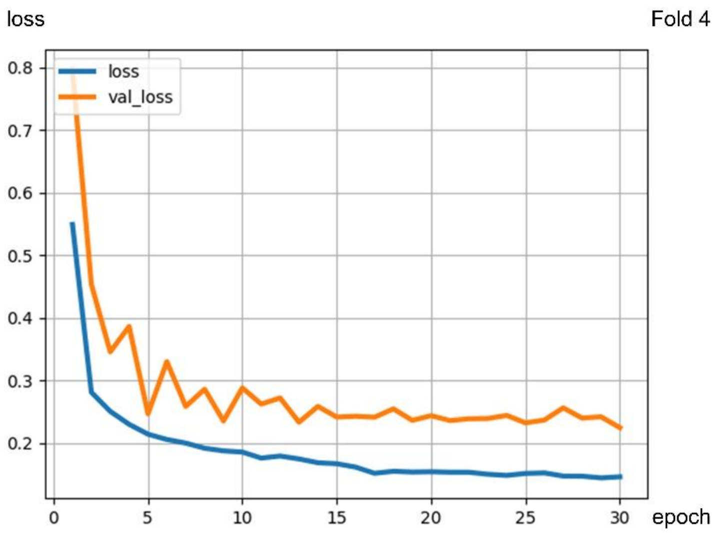

Figure S5

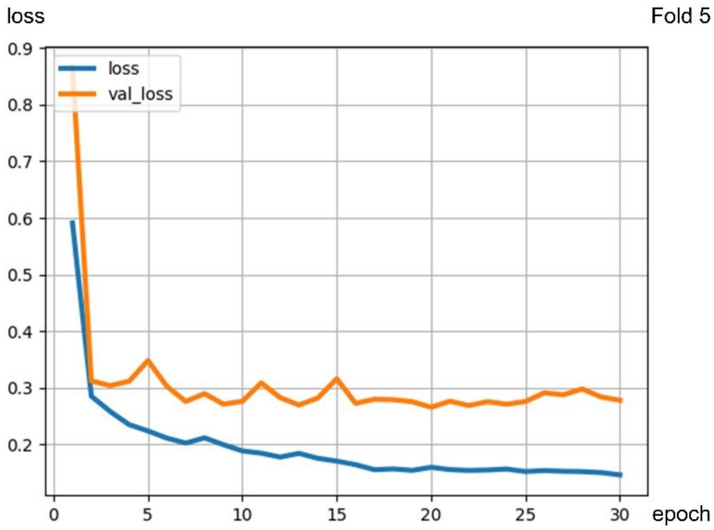
loss
Fold 5
0.9
0.8
0.7
0.6
0.5
0.4
0.3
0.2
loss
val_loss
0
5
10
15
20
25
30
epoch

Table S1: Clinical characteristics of the training and test datasets

Variable	Training dataset	Test dataset	p-value
Number of patients	180	20	
Age	59.4 ± 10.2	55.3 ± 9.7	0.09
Histological grade			1.00
Low grade	119	13	
High grade	61	7	
FIGO stage			0.54
I	125	12	
II	13	3	
III	39	5	
IV	3	0	
Deep myometrial invasion			0.63
Positive	67	6	
Negative	113	14	

Patient age is presented as the mean and standard deviation.

FIGO: International Federation of Gynecology and Obstetrics

Table S2: Magnetic resonance imaging parameters

Parameter	T1WI	T2WI	DWI	Dynamic-CE T1WI
TR (msec)	400-655	3730-7760	2300-5300/	4.7
TE (msec)	11-30	81-120	59-79	2.3
FOV (mm)	260×260	260×260	260×195-260	190-260×260
FA (degrees)	80	150	90	10
Band width, Hz/pixel	100-140	140-370	1445-2170	580
Matrix size	527×224-348	448-512×204-512	128×128	320-384×198-230
Slice thickness (mm)	4-6	4-5	4-5	5

T1WI: T1-weighted image

T2WI: T2-weighted image

DWI: diffusion-weighted image

CE: contrast-enhanced

TR: repetition time

TE: echo time

FOV: field of view

FA: flip angle

Table S3: Summary of our U-Net model

Layer (type)	Output Shape	Param	Connected to
input_1 (InputLayer)	[(None, 512, 512, 3)]	0	
conv2d_1 (Conv2D)	(None, 512, 512, 8)	224	input_1[0][0]
batch_normalization_1 (BatchNormalization)	(None, 512, 512, 8)	32	conv2d_1[0][0]
leaky_re_lu_1 (LeakyReLU)	(None, 512, 512, 8)	0	batch_normalization_1[0][0]
dropout_1 (Dropout)	(None, 512, 512, 8)	0	leaky_re_lu_1[0][0]
conv2d_2 (Conv2D)	(None, 512, 512, 8)	584	dropout_1[0][0]
batch_normalization_2 (BatchNormalization)	(None, 512, 512, 8)	32	conv2d_2[0][0]
leaky_re_lu_2 (LeakyReLU)	(None, 512, 512, 8)	0	batch_normalization_2[0][0]
dropout_2 (Dropout)	(None, 512, 512, 8)	0	leaky_re_lu_2[0][0]
max_pooling2d_1 (MaxPooling2D)	(None, 256, 256, 8)	0	dropout_2[0][0]
conv2d_3 (Conv2D)	(None, 256, 256, 16)	1168	max_pooling2d_1[0][0]
batch_normalization_3 (BatchNormalization)	(None, 256, 256, 16)	64	conv2d_3[0][0]
leaky_re_lu_3 (LeakyReLU)	(None, 256, 256, 16)	0	batch_normalization_3[0][0]

dropout_3 (Dropout)	(None, 256, 256, 16)	0	leaky_re_lu_3[0][0]
conv2d_4 (Conv2D)	(None, 256, 256, 16)	2320	dropout_3[0][0]
batch_normalization_4 (BatchNormalization)	(None, 256, 256, 16)	64	conv2d_4[0][0]
leaky_re_lu_4 (LeakyReLU)	(None, 256, 256, 16)	0	batch_normalization_4[0][0]
dropout_4 (Dropout)	(None, 256, 256, 16)	0	leaky_re_lu_4[0][0]
max_pooling2d_2 (MaxPooling2D)	(None, 128, 128, 16)	0	dropout_4[0][0]
conv2d_5 (Conv2D)	(None, 128, 128, 16)	2320	max_pooling2d_2[0][0]
batch_normalization_5 (BatchNormalization)	(None, 128, 128, 16)	64	conv2d_5[0][0]
leaky_re_lu_5 (LeakyReLU)	(None, 128, 128, 16)	0	batch_normalization_5[0][0]
dropout_5 (Dropout)	(None, 128, 128, 16)	0	leaky_re_lu_5[0][0]
conv2d_6 (Conv2D)	(None, 128, 128, 16)	2320	dropout_5[0][0]
batch_normalization_6 (BatchNormalization)	(None, 128, 128, 16)	64	conv2d_6[0][0]
leaky_re_lu_6 (LeakyReLU)	(None, 128, 128, 16)	0	batch_normalization_6[0][0]
dropout_6 (Dropout)	(None, 128, 128, 16)	0	leaky_re_lu_6[0][0]

max_pooling2d_3 (MaxPooling2D)	(None, 64, 64, 16)	0	dropout_6[0][0]
conv2d_7 (Conv2D)	(None, 64, 64, 16)	2320	max_pooling2d_3[0][0]
batch_normalization_7 (BatchNormalization)	(None, 64, 64, 16)	64	conv2d_7[0][0]
leaky_re_lu_7 (LeakyReLU)	(None, 64, 64, 16)	0	batch_normalization_7[0][0]
dropout_7 (Dropout)	(None, 64, 64, 16)	0	leaky_re_lu_7[0][0]
conv2d_8 (Conv2D)	(None, 64, 64, 16)	2320	dropout_7[0][0]
batch_normalization_8 (BatchNormalization)	(None, 64, 64, 16)	64	conv2d_8[0][0]
leaky_re_lu_8 (LeakyReLU)	(None, 64, 64, 16)	0	batch_normalization_8[0][0]
dropout_8 (Dropout)	(None, 64, 64, 16)	0	leaky_re_lu_8[0][0]
max_pooling2d_4 (MaxPooling2D)	(None, 32, 32, 16)	0	dropout_8[0][0]
conv2d_9 (Conv2D)	(None, 32, 32, 16)	2320	max_pooling2d_4[0][0]
batch_normalization_9 (BatchNormalization)	(None, 32, 32, 16)	64	conv2d_9[0][0]
leaky_re_lu_9 (LeakyReLU)	(None, 32, 32, 16)	0	batch_normalization_9[0][0]
dropout_9 (Dropout)	(None, 32, 32, 16)	0	leaky_re_lu_9[0][0]
conv2d_10 (Conv2D)	(None, 32, 32, 16)	2320	dropout_9[0][0]

batch_normalization_10 (BatchNormalization)	(None, 32, 32, 16)	64	conv2d_10[0][0]
leaky_re_lu_10 (LeakyReLU)	(None, 32, 32, 16)	0	batch_normalization_10[0][0]
dropout_10 (Dropout)	(None, 32, 32, 16)	0	leaky_re_lu_10[0][0]
max_pooling2d_5 (MaxPooling2D)	(None, 16, 16, 16)	0	dropout_10[0][0]
conv2d_11 (Conv2D)	(None, 16, 16, 16)	2320	max_pooling2d_5[0][0]
batch_normalization_11 (BatchNormalization)	(None, 16, 16, 16)	64	conv2d_11[0][0]
leaky_re_lu_11 (LeakyReLU)	(None, 16, 16, 16)	0	batch_normalization_11[0][0]
dropout_11 (Dropout)	(None, 16, 16, 16)	0	leaky_re_lu_11[0][0]
conv2d_12 (Conv2D)	(None, 16, 16, 16)	2320	dropout_11[0][0]
batch_normalization_12 (BatchNormalization)	(None, 16, 16, 16)	64	conv2d_12[0][0]
leaky_re_lu_12 (LeakyReLU)	(None, 16, 16, 16)	0	batch_normalization_12[0][0]
dropout_12 (Dropout)	(None, 16, 16, 16)	0	leaky_re_lu_12[0][0]
up_sampling2d_1 (UpSampling2D)	(None, 32, 32, 16)	0	dropout_12[0][0]
conv2d_13 (Conv2D)	(None, 32, 32, 16)	2320	up_sampling2d_1[0][0]

batch_normalization_13 (BatchNormalization)	(None, 32, 32, 16)	64	conv2d_13[0][0]
leaky_re_lu_13 (LeakyReLU)	(None, 32, 32, 16)	0	batch_normalization_13[0][0]
dropout_13 (Dropout)	(None, 32, 32, 16)	0	leaky_re_lu_13[0][0]
concatenate_1 (Concatenate)	(None, 32, 32, 32)	0	dropout_13[0][0]<br>dropout_10[0][0]
conv2d_14 (Conv2D)	(None, 32, 32, 16)	4624	concatenate_1[0][0]
batch_normalization_14 (BatchNormalization)	(None, 32, 32, 16)	64	conv2d_14[0][0]
leaky_re_lu_14 (LeakyReLU)	(None, 32, 32, 16)	0	batch_normalization_14[0][0]
dropout_14 (Dropout)	(None, 32, 32, 16)	0	leaky_re_lu_14[0][0]
conv2d_15 (Conv2D)	(None, 32, 32, 16)	2320	dropout_14[0][0]
batch_normalization_15 (BatchNormalization)	(None, 32, 32, 16)	64	conv2d_15[0][0]
leaky_re_lu_15 (LeakyReLU)	(None, 32, 32, 16)	0	batch_normalization_15[0][0]
dropout_15 (Dropout)	(None, 32, 32, 16)	0	leaky_re_lu_15[0][0]
up_sampling2d_2 (UpSampling2D)	(None, 64, 64, 16)	0	dropout_15[0][0]
conv2d_16 (Conv2D)	(None, 64, 64, 16)	2320	up_sampling2d_2[0][0]

batch_normalization_16 (BatchNormalization)	(None, 64, 64, 16)	64	conv2d_16[0][0]
leaky_re_lu_16 (LeakyReLU)	(None, 64, 64, 16)	0	batch_normalization_16[0][0]
dropout_16 (Dropout)	(None, 64, 64, 16)	0	leaky_re_lu_16[0][0]
concatenate_2 (Concatenate)	(None, 64, 64, 32)	0	dropout_16[0][0]<br>dropout_8[0][0]
conv2d_17 (Conv2D)	(None, 64, 64, 16)	4624	concatenate_2[0][0]
batch_normalization_17 (BatchNormalization)	(None, 64, 64, 16)	64	conv2d_17[0][0]
leaky_re_lu_17 (LeakyReLU)	(None, 64, 64, 16)	0	batch_normalization_17[0][0]
dropout_17 (Dropout)	(None, 64, 64, 16)	0	leaky_re_lu_17[0][0]
conv2d_18 (Conv2D)	(None, 64, 64, 16)	2320	dropout_17[0][0]
batch_normalization_18 (BatchNormalization)	(None, 64, 64, 16)	64	conv2d_18[0][0]
leaky_re_lu_18 (LeakyReLU)	(None, 64, 64, 16)	0	batch_normalization_18[0][0]
dropout_18 (Dropout)	(None, 64, 64, 16)	0	leaky_re_lu_18[0][0]
up_sampling2d_3 (UpSampling2D)	(None, 128, 128, 16)	0	dropout_18[0][0]
conv2d_19 (Conv2D)	(None, 128, 128, 16)	2320	up_sampling2d_3[0][0]

batch_normalization_19 (BatchNormalization)	(None, 128, 128, 16)	64	conv2d_19[0][0]
leaky_re_lu_19 (LeakyReLU)	(None, 128, 128, 16)	0	batch_normalization_19[0][0]
dropout_19 (Dropout)	(None, 128, 128, 16)	0	leaky_re_lu_19[0][0]
concatenate_3 (Concatenate)	(None, 128, 128, 32)	0	dropout_19[0][0]<br>dropout_6[0][0]
conv2d_20 (Conv2D)	(None, 128, 128, 16)	4624	concatenate_3[0][0]
batch_normalization_20 (BatchNormalization)	(None, 128, 128, 16)	64	conv2d_20[0][0]
leaky_re_lu_20 (LeakyReLU)	(None, 128, 128, 16)	0	batch_normalization_20[0][0]
dropout_20 (Dropout)	(None, 128, 128, 16)	0	leaky_re_lu_20[0][0]
conv2d_21 (Conv2D)	(None, 128, 128, 16)	2320	dropout_20[0][0]
batch_normalization_21 (BatchNormalization)	(None, 128, 128, 16)	64	conv2d_21[0][0]
leaky_re_lu_21 (LeakyReLU)	(None, 128, 128, 16)	0	batch_normalization_21[0][0]
dropout_21 (Dropout)	(None, 128, 128, 16)	0	leaky_re_lu_21[0][0]
up_sampling2d_4 (UpSampling2D)	(None, 256, 256, 16)	0	dropout_21[0][0]
conv2d_22 (Conv2D)	(None, 256, 256, 16)	2320	up_sampling2d_4[0][0]

batch_normalization_22 (BatchNormalization)	(None, 256, 256, 16)	64	conv2d_22[0][0]
leaky_re_lu_22 (LeakyReLU)	(None, 256, 256, 16)	0	batch_normalization_22[0][0]
dropout_22 (Dropout)	(None, 256, 256, 16)	0	leaky_re_lu_22[0][0]
concatenate_4 (Concatenate)	(None, 256, 256, 32)	0	dropout_22[0][0]<br>dropout_4[0][0]
conv2d_23 (Conv2D)	(None, 256, 256, 16)	4624	concatenate_4[0][0]
batch_normalization_23 (BatchNormalization)	(None, 256, 256, 16)	64	conv2d_23[0][0]
leaky_re_lu_23 (LeakyReLU)	(None, 256, 256, 16)	0	batch_normalization_23[0][0]
dropout_23 (Dropout)	(None, 256, 256, 16)	0	leaky_re_lu_23[0][0]
conv2d_24 (Conv2D)	(None, 256, 256, 16)	2320	dropout_23[0][0]
batch_normalization_24 (BatchNormalization)	(None, 256, 256, 16)	64	conv2d_24[0][0]
leaky_re_lu_24 (LeakyReLU)	(None, 256, 256, 16)	0	batch_normalization_24[0][0]
dropout_24 (Dropout)	(None, 256, 256, 16)	0	leaky_re_lu_24[0][0]
up_sampling2d_5 (UpSampling2D)	(None, 512, 512, 16)	0	dropout_24[0][0]
conv2d_25 (Conv2D)	(None, 512, 512, 8)	1160	up_sampling2d_5[0][0]

batch_normalization_25 (BatchNormalization)	(None, 512, 512, 8)	32	conv2d_25[0][0]
leaky_re_lu_25 (LeakyReLU)	(None, 512, 512, 8)	0	batch_normalization_25[0][0]
dropout_25 (Dropout)	(None, 512, 512, 8)	0	leaky_re_lu_25[0][0]
concatenate_5 (Concatenate)	(None, 512, 512, 16)	0	dropout_25[0][0]<br>dropout_2[0][0]
conv2d_26 (Conv2D)	(None, 512, 512, 8)	1160	concatenate_5[0][0]
batch_normalization_26 (BatchNormalization)	(None, 512, 512, 8)	32	conv2d_26[0][0]
leaky_re_lu_26 (LeakyReLU)	(None, 512, 512, 8)	0	batch_normalization_26[0][0]
dropout_26 (Dropout)	(None, 512, 512, 8)	0	leaky_re_lu_26[0][0]
conv2d_27 (Conv2D)	(None, 512, 512, 8)	584	dropout_26[0][0]
batch_normalization_27 (BatchNormalization)	(None, 512, 512, 8)	32	conv2d_27[0][0]
leaky_re_lu_27 (LeakyReLU)	(None, 512, 512, 8)	0	batch_normalization_27[0][0]
dropout_27 (Dropout)	(None, 512, 512, 8)	0	leaky_re_lu_27[0][0]
conv2d_28 (Conv2D)	(None, 512, 512, 3)	27	dropout_27[0][0]
activation_1 (Activation)	(None, 512, 512, 3)	0	conv2d_28[0][0]

==
